# Recent Advances in Practical Methods for Liver Cell Biology: A Short Overview

**DOI:** 10.3390/ijms21062027

**Published:** 2020-03-16

**Authors:** Sandra Torres, Zeinab Abdullah, Maximilian J Brol, Claus Hellerbrand, Mercedes Fernandez, Romina Fiorotto, Sabine Klein, Philipp Königshofer, Christian Liedtke, Sophie Lotersztajn, Yulia A. Nevzorova, Robert Schierwagen, Thomas Reiberger, Frank Erhard Uschner, Frank Tacke, Ralf Weiskirchen, Jonel Trebicka

**Affiliations:** 1 Department of Internal Medicine I, Goethe University Frankfurt, 60590 Frankfurt, Germany; Sandra.Torres@kgu.de (S.T.); Sabine.Klein@kgu.de (S.K.); Robert.Schierwagen@kgu.de (R.S.); Frank.Uschner@kgu.de (F.E.U.); 2 Institute of Molecular Medicine and Experimental Immunology, University Clinic, 53127 Bonn, Germany; zeinab.abdullah@ukbonn.de; 3 Department of Internal Medicine I, University Clinic, 53127 Bonn, Germany; brol@uni-bonn.de; 4 Institute of Biochemistry, Friedrich-Alexander University Erlangen-Nürnberg, 91054 Erlangen, Germany; claus.hellerbrand@fau.de; 5 Angiogenesis in Liver Disease Research Group, IDIBAPS Biomedical Research Institute, University of Barcelona, 08036 Barcelona, Spain; mercefernandez@ub.edu; 6 Yale University School of Medicine, Yale Liver Center, Department of Internal Medicine, New Haven, CT 06520, USA; romina.fiorotto@yale.edu; 7 Div. of Gastroenterology and Hepatology, Dept. of Internal Medicine III, Medical University of Vienna, A-1090 Vienna, Austria; philipp.koenigshofer@meduniwien.ac.at (P.K.); thomas.reiberger@meduniwien.ac.at (T.R.); 8 HEPEX Lab for Liver Fibrosis and Portal Hypertension, Medical University of Vienna, A-1090 Vienna, Austria; 9 Department of Internal Medicine III, University Hospital RWTH Aachen, 52074 Aachen, Germany; cliedtke@ukaachen.de (C.L.); ynevzorova@ukaachen.de (Y.A.N.); 10 Inserm UMR-1149, Centre de Recherche sur l’Inflammation, 75890 Paris, France; sophie.lotersztajn@inserm.fr; 11 Sorbonne Paris Cité, Laboratoire d’Excellence Inflamex, Faculté de Médecine, Site Xavier Bichat, Université Paris Diderot, 75018 Paris, France; 12 Département Hospitalo-Universitaire UNITY, Service d’Hépatologie, Hôpital Beaujon, Assistance Publique-Hôpitaux de Paris, 92110 Clichy, France; 13 Department of Genetics, Physiology and Microbiology, Faculty of Biology, Complutense University Madrid, Spain; 12 de Octubre Health Research Institute (IMAS12), 28040 Madrid, Spain; 14 Department of Hepatology & Gastroenterology, Charité University Medicine Berlin, 10117 Berlin, Germany; frank.tacke@charite.de; 15 Institute of Molecular Pathobiochemistry, Experimental Gene Therapy and Clinical Chemistry, (IFMPEGKC), University Hospital Aachen, D-52074 Aachen, Germany; rweiskirchen@ukaachen.de; 16 European Foundation for Study of Chronic Liver Failure, 08021 Barcelona, Spain; 17 Institute of Bioengineering Catalunya, 08028 Barcelona, Spain; 18 University of Southern Denmark, 5230 Odense, Denmark

**Keywords:** hepatic stellate cells, hepatocellular cancer, fibrogenesis, steatosis, in vitro models

## Abstract

Molecular and cellular research modalities for the study of liver pathologies have been tremendously improved over the recent decades. Advanced technologies offer novel opportunities to establish cell isolation techniques with excellent purity, paving the path for 2D and 3D microscopy and high-throughput assays (e.g., bulk or single-cell RNA sequencing). The use of stem cell and organoid research will help to decipher the pathophysiology of liver diseases and the interaction between various parenchymal and non-parenchymal liver cells. Furthermore, sophisticated animal models of liver disease allow for the in vivo assessment of fibrogenesis, portal hypertension and hepatocellular carcinoma (HCC) and for the preclinical testing of therapeutic strategies. The purpose of this review is to portray in detail novel in vitro and in vivo methods for the study of liver cell biology that had been presented at the workshop of the 8th meeting of the European Club for Liver Cell Biology (ECLCB-8) in October of 2018 in Bonn, Germany.

## 1. Introduction

**Figure 1:**
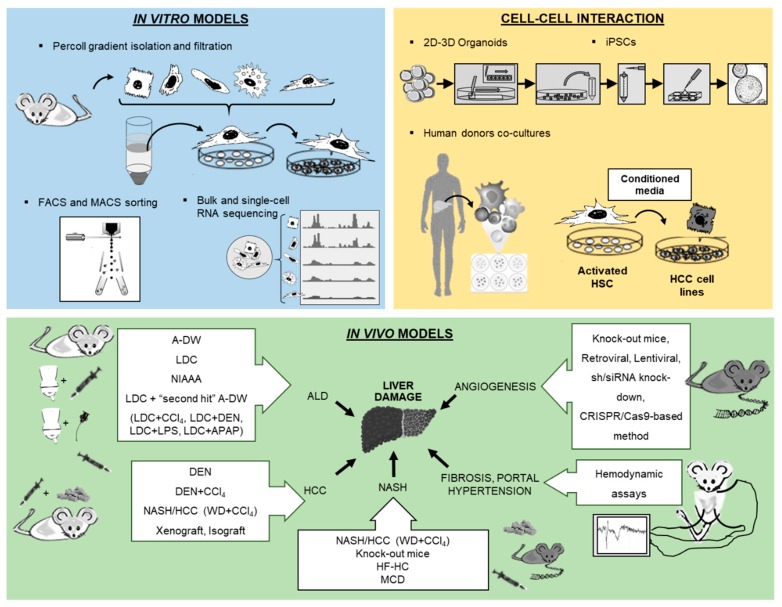
Overview of new methodologies using in vitro, cell–cell interaction and in vivo models for the study of liver pathology.

Cell culture techniques are important tools for the study of pathogenesis and treatment of liver diseases. Each different cell type plays a specific role in the liver. The parenchymal cells, mainly hepatocytes, constitute 80% of the total liver volume and are responsible for the majority of liver functions. The nonparenchymal cells include hepatic stellate cells (HSCs), liver sinusoidal endothelial cells and resident macrophages such as Kupffer cells (KCs) **[1]**. The connective tissue of the liver consists of blood vessels, nerves, lymphatic vessels, bile canaliculi and extracellular matrix [2]. Inside the bile canaliculi, the epithelial cells, the so-called cholangiocytes, participate in the regulation of bile production and in the process of biliary repair [3]. In recent years, biliary research and cholangiocytes gained much interest in the research of liver diseases. Although conventional procedures to isolate liver cells, such as in situ liver perfusion and collagenase digestion followed by density gradient isolation, are well established, novel methods have been introduced such as fluorescence-activated cell sorting (FACS) of HSCs using vitamin A autofluorescence. These approaches provide high purity of HSCs and are reproducible. In the coming years, the use of 2D microscopy, 3D microscopy, intravital microscopy, flow cytometry and cell isolation for subsequent functional experiments or expression analyses is expected to significantly increase characterization of hepatic cell populations and their interactions with circulating immune cells. For instance, these methods have been used to study the impact of hepatic macrophages and monocytes on steatosis, inflammation, hepatocellular injury, HSCs activation and angiogenesis [4]. Two novel technologies from stem cell research, induced pluripotent stem cells (iPSCs) and liver organoids, helped to decipher the pathophysiology of cholangiopathies [5]. Using in vitro models of primary human liver cells from different human donors, co-culture systems can simulate additional pathophysiological liver cell interactions, being complementary to animal models in preclinical analysis. Furthermore, development of animal models to experimentally mimic alcoholic liver disease (ALD), such as the liquid Lieber–DeCarli (LDC) diet, intragastric ethanol infusion and administration of carbon tetrachloride (CCl_4_) combined with ethanol in drinking water, can help to understand critical pathophysiological steps of human ALD, such as inflammation and fibrosis during ALD progression [6,7]. Moreover, HCC models are receiving increased attention, not only for the fast xenograft model, but also for application of *N*-nitrosodiethylamine (DEN) followed by repeated administration of CCl_4_ (Table 1) [8], as well as the model of non-alcoholic steatohepatitis (NASH) combined with a HCC model described recently by using a Western diet (WD) combined with CCl_4_ (Table 1) [7,9]. Finally, the assessment of systemic, splanchnic and portal hemodynamics in animal models is now well accepted and crucial for the evaluation of drugs for end-stage liver disease. The purpose of this review is to present technological tools that will enhance our ability to isolate and purify the different cell populations and other strategies to study the full spectrum of liver disease (Figure 1).

## 2. In Vitro Models

### 2.1. Isolation and Culture of Hepatocytes

Hepatocytes are the major cell population in the liver [57]. Hepatocytes are responsible for management of nutrient uptake, blood detoxification and packaging and secretion of proteins, lipids and bile [58]. Their isolation was well established by the two-step EGTA/collagenase perfusion technique by Seglen in 1976 [59]. The 2D culture of primary hepatocytes is considered the “gold standard” for in vitro testing of the hepatotoxic effect of therapies and drug metabolism [60]. The disadvantage of 2D culture is that within a few days, hepatocytes suffer dedifferentiation and loss of function. The 3D techniques are gaining relevance due to the presence of the non-parenchymal cells that play the role of maintaining hepatocyte functions [28]. Developments in the production of 3D hepatocyte culture scaffolds are helpful in understanding the cross talk between hepatocytes and non-parenchymal cells and their role in the regulation of liver pathologies.

Cellular stress during the isolation techniques results in a low quality of isolated cells and reduced cell engraftment after transplantation. The use of 3D cell formats has also been successful in processes of cryopreservation and post-thawing, improving in vivo efficacy [28]. Furthermore, applying good manufacturing practices (GMPs) improves hepatic functions and the ability to engraft in vivo, compared to the classic organ storage solution based on the University of Wisconsin medium [61].

Hepatocytes-like cells derived from subsequent differentiation of iPSCs using a cocktail of growth factors and specific matrices is another alternative technique to obtain hepatocytes in vitro [24,25,62]. One disadvantage is that iPSC-derived hepatic cells are phenotypically more similar to fetal hepatocytes than to the freshly isolated counterpart [63]. To overcome this problem, new strategies include a stepwise induction with cocktails of small molecules to improve the final maturation [26,64].

### 2.2. HSCs Isolation and Immortalized HSCs Lines

HSCs, the main vitamin A-storing cells located in the perisinusoidal space between hepatocytes and sinusoids, play a key role in collagen production, secretion and function of cytokines and chemokines, as well as in the modulation of the immune system in addition to changes in contractile features during homeostasis and liver fibrosis [65]. It has now become clear that not all functions are done by the same HSC, but that the transcriptional profile of single HSCs considerably varies after activation in vivo, particularly during fibrogenesis [4]. First protocols for HSC isolation were established in the 1980s, with collagenase/pronase digestion of the liver tissue and subsequent fractioning process of the heterogeneous cell suspension on a density gradient [66]. In 1998, other methods to isolate HSCs appeared, such as the first protocol to isolate HSCs with FACS. This involved applying FACS to sort cells from rats with high purity, using simply their high side scatter of incident light (Table 1) [10]. Later, FACS instruments equipped with a UV laser could sort HSCs by visualizing the typical autofluorescence from vitamin A storage (Table 1) [11,12].

HSC isolation with FACS sorting allows isolation with high purity, and, compared to standard techniques, FACS-based protocols can be used to isolate HSCs from much younger animals, such as genetically modified mice characterized by a short life span. Moreover, these protocols improve isolation of HSCs when hepatocytes are fattened in steatosis models. In addition, these protocols permit simultaneous comparison of hepatic cell subpopulations from the same animal. Despite these advantages, FACS sorting requires investment in special equipment, including UV lasers, appropriate filters and specific skill acquisition. Furthermore, these protocols require pooling of livers, long time periods for cell sorting and careful use of UV light, which can be stressful and cause cell damage.

In summary, HSC sorting by FACS via vitamin A autofluorescence provides the opportunity to obtain excellent cell purity and, despite the fact that isolated HSCs cannot reproduce the same phenotype as the one found in cirrhotic livers, these protocols can be used to optimize the study of HSCs biology and their role in liver fibrosis. Undeniably, the co-culture systems of HSCs with other cells could explain the relevant role of cell–cell interactions and the paracrine influences. This has been exemplarily demonstrated more than a decade ago for the co-culture of HSC with Kupffer cells that mimicked the in vivo activation of HSC much more accurately than single HSC cultures [67].

### 2.3. Analysis of Liver Cells from Different Human Donors and Co-Culture Models

Despite the emergence of therapeutic targets for liver pathologies, the response of the treatments shows significant variations between patients. In vitro studies with liver cells from different human donors could help to understand and determine the differences observed in in vivo studies. Use of complex co-culture models could be a start to better simulate the complexity of the in vivo situation. This is currently intensively explored for fatty liver disease conditions, in which novel 3D biochip systems partially allow modelling complex cellular interactions between steatotic hepatocytes and non-parenchymal cells [68]. However, for the comparison of primary liver cells from different human donors, it is critical to exclude that isolation procedures can affect functional cellular characteristics. Therefore, it is important that it has been shown that different HSC isolation procedures from tissue of the same donor resulted in no significant differences, whereas HSCs isolated from different human donors revealed significant variations (Table 1) [16]. The differences in the expression levels of profibrogenic genes observed between HSCs from different human donors [69] may reveal some aspects of varying fibrosis progression in patients as well as development of HCC.

Furthermore, numerous studies have shown that HSCs are related to the formation and progression of HCC (Table 1) [17,18]. The treatment of human HCC cells with conditioned media of HSCs from different human donors resulted in significantly different functional effects as well as gene expression changes in the HCC cells [70]. Bioinformatic modeling led to the identification of pregnancy-associated plasma protein A (PAPPA) as novel cancer-promoting stromal factor secreted by HSCs, which is related to advanced-stage HCC [70]. In addition, HSCs can also be used in co-culture models with other cells, such as HSCs treated with conditioned media from different human melanoma cells (Table 1) [19], or conditioned media from steatotic human hepatocytes that produce a more fibrotic phenotype in HSCs compared with normal hepatocytes (Table 1) [20].

In summary, in vitro (co-)culture models of primary human liver cells from different human donors can be a valuable system to simulate at least certain aspects of the heterogeneity and variation of the course of chronic liver disease. Furthermore, in vitro studies with human liver cells may be used to predict the response of patients prior to defined therapies.

### 2.4. Isolation and Characterization of Liver Macrophages

Liver macrophages play a key role in innate immunity, homeostasis and inflammation. Among liver macrophages, two principal populations can be differentiated by their ontogeny, polarization and function during injury and resolution [71], namely KCs and monocytes, and their functional behavior in liver diseases could become a target for novel therapeutics (Table 1) [38]. Liver macrophages have been characterized by means of new technologies, such as multicolor flow cytometry, advanced microscopy, sorting followed by bulk sequencing or single-cell RNA sequencing (Table 1) [4,13,21,22,23,72]. From an isolation perspective, KCs are firmly attached to sinusoidal endothelial cells and require gentle perfusion-based dissociation methods [73], while monocytes can be isolated simply by collagenase digestion (Table 1) [40]. These protocols are based on perfusion of the liver via portal vein cannulation, cell extraction, purification via density gradient or sedimentation and optional additional steps for cell sorting based on FACS or MACS. The inconvenience of these techniques lies in the activation of hepatic macrophages during isolation and the fact that they also require adaptation based on the mouse strains used, making cross-lab standardization difficult. Moreover, compared to the MACS sorting method, FACS usually results in excellent purity and can be flexibly adjusted to the populations of interest. However, FACS can impact cell viability, it is time-consuming and expensive. Similar to HSC, macrophages behave differently in vitro, if cultured alone, together with other cells or in the context of the liver structure with zonation, flow conditions and nutrient / oxygen gradients.

Many studies are currently performed to understand human liver macrophages. Initial reports using single-cell RNA sequencing confirmed the heterogeneity and functional diversification of macrophages in healthy and diseased human livers [23,72]. However, important pitfalls include the limited availability of human samples, ischemic alteration of tissue during surgical procedures, heterogeneity of patients and the relative paucity of specific markers for hepatic macrophage subsets in humans [71]. For instance, the c-type lectin “clec4f” is considered specific for KCs in mice, but it has no direct counterpart in humans [74,75].

### 2.5. Cellular Models in Biliary Research

Cholangiocytes are epithelial cells of the bile ducts. Their function is to modify the composition and volume of bile produced by hepatocytes en route to the duodenum. Different sub-populations of cholangiocytes exist: the large cholangiocytes involved in secretory processes and the smaller cholangiocytes equipped with plasticity to proliferate in response to damage [76]. Cholangiopathies represent a significant cause of liver-related morbidity and mortality and are an important indication for liver transplantation [77].

Cholangiocyte isolation is realized firstly by in situ liver perfusion and collagenase digestion, secondly by mechanical and enzymatic digestion, and finally the cells are separated by filtration. At this point, it is possible to obtain different sizes of cells, intrahepatic bile duct units (IBDUs) (Table 1) [14] or, via immune-magnetic separation, biliary epithelial cells (BECs) (Table 1) [15]. While IBDU isolation is not completely pure, it is sufficient for secretory function studies 48 h after plating on matrigel [78,79,80]. In contrast, BEC can be more purified but cannot proliferate in culture. Recently, it has been shown that primary cultures of mouse cholangiocytes could be cultured on rat tail collagen for several passages [81] and used for specific functional studies with transgenic mice [82].

Cholangiocytes from human liver explant tissue can also be isolated by collagenase digestion followed by Percoll gradient isolation and immune magnetic positive selection and can then be expanded in culture since they acquire a more mesenchymal phenotype [83]. Furthermore, it is possible to culture cholangiocytes from a small fragment of a liver biopsy, but with a lower purification [84]. Surprisingly, these cholangiocyte phenotypic markers are established: γ-glutamyl-transpeptidase (GGT), cytokeratins 7 and 19, EpCam, SOX-9, secretin receptor and cystic fibrosis transmembrane conductance regulator (CFTR) [85].

The study of human cholangiocytes is relevant to the understanding of the biological functions of the biliary epithelium and cholangiopathies. Despite relentless efforts to isolate cholangiocytes, the main challenge lies with the yield and the purity of the cell preparation technique since these represent about 4% of the total liver cell populations, similar in size to endothelial and KCs. Other limitations are the need of a polarized organization to improve their functionality, the time-consuming method and the complex media needed for expansion.

Compared to other liver cell isolation models, accessibility to human tissue is limited and restricted to the end stage of liver disease. In the case of animal models, there are physiological inter-species differences. A number of other options are currently under investigation, such as iPSCs and the possibility to isolate 3D liver organoids, which may provide opportunities to overcome current limitations (Table 1) [27].

## 3. In Vivo Models

### 3.1. Angiogenesis and Gene Expression Inhibition

One of the hallmarks of chronic and liver disease is angiogenesis with new formation of blood vessels from preexisting vasculature [29,86]. In this pathological condition, abnormal angioarchitecture is established together with fibrogenesis, inflammation and tumorigenesis, resulting in the formation of portosystemic collateral vessels, an increase in splanchnic blood flow and the aggravation of portal hypertension. All of these could be targets for new therapeutic approaches in treating angiogenesis in chronic liver disease (Table 1) [29,86].

To address angiogenesis, a number of different methods have been developed in recent decades [87]. Using in vitro bioassays, purified endothelial cell cultures or co-cultures with supporting cells (e.g., smooth muscle cells, pericytes, fibroblasts and tumor cells), either on 2D monolayers or 3D spheroids, were employed to mimic aspects of in vivo vascular formation. However, there are some critical points to consider regarding the in vitro assays and the choice of endothelial cells. Different factors characterize endothelial cells: they have species- and organ-associated phenotypic differences and depend on their microvascular origin. Also, the number of passages, cells plated per well and the use of growth factor-reduced matrigel are factors influencing the success of these assays. The use of primary cells derived from inducible knock-out mice, or the use of cells genetically altered by retroviral or lentiviral constructs, including fluorescence (gain-of-function) or by shRNA/siRNA knock-down or genome-editing CRISPR/Cas9-based methods (loss-of-function), has provided answers to specific hypotheses in angiogenesis processes. As opposed to in vitro assays, using in vivo assays, these hypotheses can be validated by immunohistochemical, histological and molecular biology procedures. Recently, the use of angiogenesis inhibitors in vivo has shown a potential effect in chronic liver disease, such as inhibiting the VEGF signaling pathway, combining treatments with VEGF and PDGF inhibitors directed against endothelial cells and pericytes, or gene therapy with cell-targeted molecule-targeted liposomal small interference RNAs (Table 1) [53,88,89]. Endogenous angiogenesis inhibitors using adenovirus-mediated gene transfer are also applied (Table 1) [56,90]. Gene expression inhibition studies employing in vivo loss-of-function models have shown that post-transcriptional mechanisms, regulated by cytoplasmic polyadenylation element binding proteins (CPEB), are essential for pathological angiogenesis in chronic liver disease, but dispensable for homeostasis of healthy vessels and physiological angiogenesis (Table 1) [30,54,55].

Despite the fact that in angiogenesis studies in vitro assays can be faster and reproducible, in vivo assays are physiologically more relevant. Moreover, in vitro assays do not represent the same conditions and do not allow study of the complex physiological interactions that occur in vivo. However, an in vivo angiogenic response also presents inconveniences, as it is a time-consuming and costly method, and it influences other parallel processes such as fibrogenesis and inflammation. Furthermore, angiogenesis image analysis requires advanced skills to avoid inter-observer variability. Finally, in vitro and in vivo models are essential to identify potential targets in neovascular processes in order to apply in new therapies in patients with chronic liver disease.

### 3.2. The Leading Models of Experimental ALD

ALD is one of the main causes of liver disease. The spectrum includes simple steatosis to cirrhosis, and it can lead to the development of end-stage HCC [91]. Unfortunately, there is no effective therapy. For the development of novel therapies, experimental animal models can provide more understanding of the mechanisms involved. The following is a short overview of the top three classic ALD experimental models and their hallmarks. First, several factors must be taken into account that could influence ALD models (Table 1) [31,92], such as gender, genetic background and age of mice. While female mice can develop alcoholic liver injury faster, they are less likely to progress to cirrhosis and HCC. For ALD models, the C57BL/6NCrl strain, due to its metabolism, is the most suitable. Moreover, the age recommended for onset of ALD models in mice is 8–11 weeks with a body weight above 19 g.

A-DW is a model for alcohol consumption in rodents, whereby the concentration of EtOH in the drinking water is gradually increased, and thereafter the animals are kept on the highest concentration throughout the study (up to 25% *v*/*v*, from eight up to 70 weeks). Other factors can be modified, such as opting to choose water or alcohol, or using multiple bottles with different alcohol concentrations or drinking in the dark. Despite the fact that this model is physiological, inexpensive, without significant mortality and with very simple animal husbandry, the strong natural aversion to alcohol of the animals results in reduced consumption and produces a reduced blood alcohol concentration (BAC) (50–70 mg/dL), thus inducing only moderate, clear steatosis and low elevations of ALT and AST without signs of fibrosis or inflammation [92,93].

The LDC diet is a liquid diet to which EtOH is added. This diet also contains necessary nutrients. At first, the EtOH is increased gradually from 1% to a concentration of 5.07% w/v (6.4% *v*/*v*) over a period of seven days. Next, mice are maintained with the highest EtOH concentration during a period which normally varies from 4 to 12 weeks. The control animal group is fed with LDC with the same isocaloric conditions as the LCD-EtOH diet. This diet has the advantage of being more time-efficient resulting in BAC ranging from 100 to 160 mg/dL. Preparation and management are straightforward, without specific equipment requirements and may be easily approved by local ethical committees. However, animals drink this diet when they are hungry and thirsty, and it is thus not completely physiological. The diet is freshly prepared every day, and the animals must be monitored during the entire process. As with AD-W, this diet also produces a mild elevation of serum transaminases and does not mimic advanced stages of human ALD such as cirrhosis and HCC (Table 1) [31,32].

There are different combinations of the LDC diet with secondary hepatic stressors or “second hits”, which have been widely used, producing models of progressive ALD. These combinations include the NIAAA model using 5% *v*/*v* LCD diet for ten days or eight weeks + single or multiple EtOH binges (5 g/kg) [33], the fibrotic model using moderate 2% LDC + CCl_4_ (1µL/g body weight, intraperitoneally (i.p.)), twice a week) (Table 1) [34], the HCC model with LCD (7–10 weeks) + DEN (40–100 mg/kg i.p.) [84], the LDC diet (8–10 weeks) + LPS (small-dose (1µg/g body weight) or high-dose (0.5 mg/kg, body weight)) [53] and the drug-induced liver injury model with the combination of LDC diet (4–6 weeks) + APAP (0.5–1 g/kg i.p.) [84].

In the intragastric ethanol infusion (IEI) method, mice are directly connected to an infusion pump with a catheter implanted into the stomach under aseptic conditions. Alcohol is added to the LDC diet and administered to the mice for a minimum period of six months. This method has the advantage of a sustained high BAC (250–500 mg/dL) and total control of nutritional intake. Furthermore, it can successfully produce advanced human ALD with the characteristic steatosis, apoptosis, central necrosis, inflammation, portal and bridging fibrosis. Nevertheless, this model bears the risk of infection and irritation, sometimes associated with dysbiosis. Therefore, it requires a high skill in implantation and considerable investment in equipment, with consequent difficulties in obtaining authorization from the local ethic committees [6,92].

In brief, none of the above-mentioned animal models can reproduce the features of human ALD due to the animals’ strong natural aversion to alcohol, high basal metabolic rate, fast catabolism of alcohol, spontaneous reduction in alcohol intake when acetaldehyde blood levels increase and absence of addictive behavior [94]. Nevertheless, these models can provide useful insights for novel therapeutic strategies.

### 3.3. Mouse Models for Non-Alcoholic Fatty Liver Disease

Non-alcoholic fatty liver disease (NAFLD) is the pathology induced in the liver characterized by fat deposition and hepatocyte steatosis. NASH is one of the stages in the spectrum of NAFLD that can progress to fibrosis, cirrhosis and, finally, to HCC. NASH is characterized by hepatocellular ballooning with fat vacuoles and the presence of inflammatory infiltrates. Unfortunately, as in other liver pathologies, not all animal models can replicate the full spectrum of human NASH. In order to study the mechanisms produced in NASH pathology and the potential therapeutic targets, the models most commonly used are as follows [42]: dietary models, such as HFD, high-fructose diet [43], cholesterol and cholate diet [37], MCD diet [95] and choline-deficient l-amino acid-defined (CDAA) diet [96]; genetic models, some of which act by promoting fat synthesis (leptin deficiency ob/ob mice [44], leptin receptor deficiency db/db mice or fa/fa in rat models [36,42]), while others act by inhibiting lipid peroxidation (peroxisome proliferator-activated receptor-α knock-out mice) [97] and impeding fat transport (ApoE knock-out mice) [35]; and chemical models, such as CCL4 [98], tetracycline [99] and streptozotocin in combination with HFD [100]. In brief, these models can reproduce different aspects of human NASH.

### 3.4. Mouse Models for Hepatocellular Carcinoma

HCC is the fifth leading cancer worldwide. Despite medical advances, treatment options are limited. HCC primarily occurs in the setting of chronic liver injury in a multistep process involving hepatitis (often associated with steatosis), liver fibrosis and cirrhosis. Animal models to mimic the different stages of human HCC are performed according to European/international animal welfare regulations as outlined elsewhere [101]. These models can be categorized into cell transplantation models, genetic models or chemical models alone or in combination with a second hit.

Cell transplantation HCC models consist of injection of hepatoma cells into recipient mice, either orthotopic in the liver (through intrahepatic or intrasplenic injection) or ectopic through subcutaneous application. Immune-deficient “nude-mice” are used for xenograft transplantation, enabling any kind of cell transplantation. Isograft transplantation consists of injection of hepatoma cells into recipient mice with identical genetic backgrounds. These models are fast and inexpensive, with easy management of the hepatoma cells before transplantation and without invasive monitoring of tumors. They are suitable for the testing of new drugs in HCC therapy. Nevertheless, these models do not allow study of all stages of progression of HCC, and in some models of tumors are occasionally rejected.

As a result of technological advances, genetic HCC mouse models, i.e., genetically engineered animal models of HCC, are increasingly developed [102]. Some of them enable study of steatosis, inflammation and fibrosis, such as liver-specific nuclear factor (NF)-κB essential modulator knock-out mice (NEMO) [103] or constitutive Mdr2-/- mice [104]. Patent regulations and material transfers are limitations of these models.

Of the genotoxic carcinogens, the DEN model of liver cancer is probably the best established chemical HCC model (Table 1) [45], whereby a single DEN injection in male mice at the age of exactly 14 days will give rise to small neoplastic lesions after 22 weeks and multinodular HCC after 40 weeks. The DEN injection model provides a high tumor incidence of 90%–100% and is easy to evaluate quantitatively by simply counting number and size of developed HCC nodules. As with other HCC models, the DEN chemical model cannot mimic the entire disease progression as in humans and testing of expensive new drugs is time-consuming. The DEN chemical model can also be complemented with a weekly injection of hepatotoxins, CCl_4_, as a second hit (Table 1) [46,47]. This model can mimic the human HCC processes, such as inflammation and fibrosis, after 30 days. However, it requires more effort, and measurement of tumors can be challenging due to changes in liver architecture.

The NASH/HCC model, a combination of NASH and weekly low-dose CCl_4_ injections (Table 1) [48], can reflect the progression of human fatty liver disease from simple steatosis to cancer within 24 weeks. With a high incidence within a short period of time, this model shows a close transcriptome similarity to human non-alcoholic fatty liver disease.

All these models provide important translational relevance, as they can mimic some of the human HCC mechanisms. For example, the c-Myc transgenic animal model is highly similar to human HCC with good prognosis, while DEN-derived tumors reflect human HCC in patients with poor survival (Table 1) [9,49]. Moreover, the NASH/HCC model develops similar mechanisms of human NAFLD) [48]. In brief, these models can be most valuable in the prognosis of chronic liver disease patients and thus decrease HCC incidence and mortality.

### 3.5. In Vivo Setup of Liver Disease Models for Surgery and Hemodynamic Assessments

Cirrhosis and portal hypertension cause significant morbidity and mortality [105]. Non-selective betablockers are currently the only medical therapy for portal hypertension, but have limited efficacy [106], and do not decrease liver fibrosis and intrahepatic vascular resistance. Thus, more effort is required to identify novel antifibrotic or anti-portal hypertensive drugs.

Importantly, the study of hepatic hemodynamics in animal models within the EU should follow the EU directive 2010/63/EU and national regulations [107,108]. Important recommendations for animal health monitoring, routine laboratory animal activities and animal welfare were specified by the Federation for Laboratory Animal Science Associations (FELASA) [109]. Furthermore, the principle of the 3Rs of Russell and Burch [110] must be considered in research with animals.

The appropriate handling of animal models of liver disease and the hemodynamic measurement requires experience and regular monitoring of the general health condition, such as of jaundice, ascites or hepatic encephalopathy during the induction period [39]. 

At evaluation of portal hypertension in vivo, the appropriate choice of anesthesia is another important aspect as it impacts on arterial blood pressure, heart rate (HR) and temperature. The equipment for anesthesia monitoring should include pulse oximeter, ECG and rectal temperature probe. The choice of anesthetics is based on pharmacodynamic characteristics, options for antagonization, administration routes and differences in sensitivity related to sex, animal species and strain [111]. In addition, the planned interventions, such as vascular cannulations must be taken into consideration, which can be surgically challenging and require specific hemodynamic equipment [51]. A typical hemodynamic assessment of the portal hypertensive syndrome requires about 45 min per animal for the simultaneous acquisition of several important systemic, splanchnic and portal hemodynamic parameters.

A single injection of anesthesia requires less equipment, is relatively cheap, and does not require extensive skill with animal handling. However, the hemodynamic assays described below are very sensitive to the depth of anesthesia, which is challenging by a single injection and moreover could interfere with the results of the measurements. In contrast, the combination of inhalation anesthesia supported by intubation and injective anesthetics allows for an improved control and safety of the procedures.

For a detailed evaluation of the various hemodynamic parameters characterizing the portal hypertension syndrome and some other surgical interventions, using mice may represent a limitation due to their small body size that requires a high level of expertise and degree of technical expertise. This is probably the most important reason why usually rats are used when several hemodynamic parameters must be simultaneously obtained.

The main hemodynamic parameters to characterize the portal hypertension syndrome are portal pressure (PP), mean arterial pressure (MAP), HR, superior mesenteric artery blood flow (SMABF) and portal vein blood flow (PVBF). The ratio between MAP and HR represents a hyperdynamic index, which reflects the severity of hyperdynamic circulation caused by peripheral/splanchnic vasodilation. MAP/HR are usually invasively measured via cannulation of the femoral or carotid artery following surgical dissection and using an intravascular catheter connected to a pressure transducer. After median laparotomy, SMABF can be measured by perivascular, non-constrictive ultrasound flow probes at the superior mesenteric artery as a surrogate parameter for splanchnic vasodilation. A similar, but different-sized flow probe is available for measurement of PVBF at the portal vein. Importantly, flow probes must be calibrated for the viscosity of blood, and their size has to be chosen according to the targeted vessel size. Portal pressure as main readout parameter is assessed by a direct cannulation of a suitable mesenteric venous blood vessel, usually at the junction of the ileocolic vein by an intravascular catheter. Portal pressure is recorded via a pressure transducer after careful advancement of the catheter to the portal vein close to the liver hilum. Intrahepatic vascular resistance can be calculated by PP and PVBF.

In patients, portal pressure can also be measured invasively via the puncture of the portal vein; however, nowadays, mostly the indirect measurement via the hepatic venous pressure gradient (HVPG) assessed via transjugular liver vein catheterization is used. Invasive assessment of arterial pressure and HR is also possible via an arterial line as in an intensive care unit. In addition, splanchnic and portal blood flow may be semi-quantitatively assessed by percutaneous Doppler ultrasound. Novel dynamic contrast-enhanced CT- or MRI-based cross-sectional imaging methods are currently being developed that may allow for a quantification of blood flow in various splanchnic and portal-venous and arterial blood vessels.

## 4. Conclusion

Advanced technologies in molecular and cellular research have rapidly evolved to meet the demands of clinical applications involving diagnostics and therapeutics in liver diseases.

**Figure 2:**
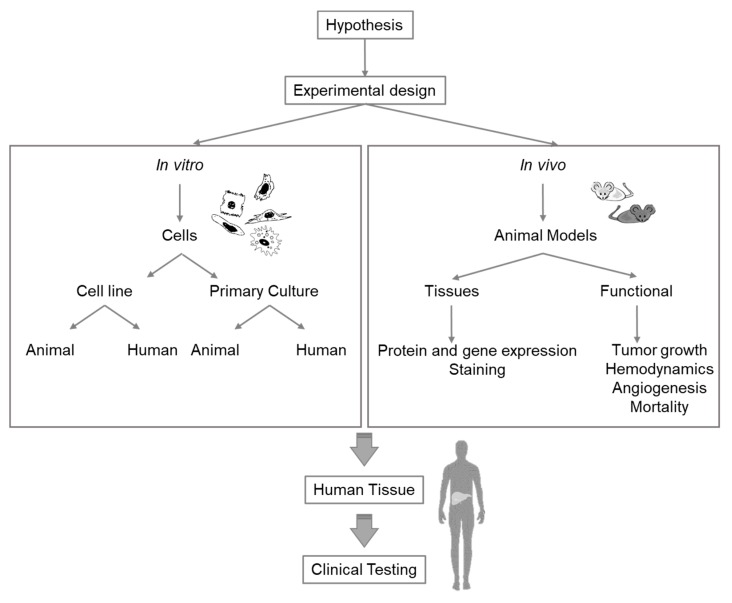
Diagram of considerations for selection of the appropriate experimental model for liver research.

Extensive research is required to select the appropriate model for specific liver research issues. Any experiment or model for research purposes must be meticulously planned and should combine different in vivo and in vitro models (Figure 2). While the currently existing knowledge gaps in the study of liver pathophysiology can be addressed with all these methodologies, future techniques and methods for different fields must, nevertheless, be continuously adapted for liver research (Supplementary Table S1).

## Figures and Tables

**Table 1:** Select findings of new methodology applications in in vitro organoids and in vivo models.

Model	Method	Application	References
In vitro	FACS and MACS sorting	HSC	[10,11,12]
		Macrophages (KCs and monocytes)	[13]
	Percoll gradient isolation and filtration	Cholangiocytes	[14,15]
	Human donor co-cultures	HSC	[16,17,18,19,20]
		Hepatocytes	[20]
	Bulk sequencing and single-cell RNA	Macrophages (KCs and monocytes)	[13,21,22,23]
	iPSCs	Hepatocytes	[24,25,26]
		Cholangiocytes	[27]
Organoids	3D liver organoids	Hepatocytes	[28]
		Cholangiocytes	[27]
	2D and 3D spheroids for angiogenesis	Endothelial cells Angiogenesis	[29,30]
In vivo	A-DW, LDC, NIAAA LDC + “second hit” (LDC + CCl_4_, LDC + DEN, LDC + LPS, LDC + APAP)	Alcoholic liver disease	[6,7,31,32,33,34]
	NASH/HCC (WD + CCl_4_) Knock-out mice HFD HF-HC MCD	Non-alcoholic liver disease	[1,7,9,35,36,37,38,39,40,41,42,43,44]
	Xenograft Isograft DEN DEN + CCL_4_ NASH/HCC (WD + CCl_4_)	Hepatocarcinoma	[7,9,45,46,47,48,49]
	Hemodynamic assays Monitoring protocols HVPG CT- or MRI-based cross imaging	Portal hypertension	[39,50,51,52]
	Knock-out mice, retroviral, lentiviral, sh/siRNA knock-down, CRISPR/Cas9-based method	Angiogenesis	[53,54,55,56]

A-DW: alcohol in drinking water; APAP: acetaminophen; HF-HC: high fat/high cholesterol; HFD: high-fat diet; LPS: lipopolysaccharide; MACS: magnetic-activated cell sorting; MCD: methionine choline deficient; NIAAA: mouse model of chronic and binge ethanol feeding.

## Supplementary Materials

Supplementary materials can be found at https://www.mdpi.com/1422-0067/21/6/2027/s1.

## Abbreviations

A-DW	Alcohol in drinking water
ALD	Alcoholic liver disease
APAP	Acetaminophen
BAC	Blood alcohol concentration
BEC	Biliary epithelial cells
CCL_4_	Carbon tetrachloride
CDAA	Choline-deficient l-amino acid-defined
CFTR	Cystic fibrosis transmembrane conductance regulator
DEN	*N*-nitrosodiethylamine
FACS	Fluorescence-activated cell sorting
FELASA	Federation for Laboratory Animal Science Associations
GGT	Γ-Glutamyl-transpeptidase
HCC	Hepatocellular carcinoma
HFD	High-fat diet
HR	Heart rate
HSCs	Hepatic stellate cells
HVPG	Hepatic venous pressure gradient
I.m.	Intramuscularly
I.p.	Intraperitoneally
IBDU	Intrahepatic bile duct units
IEI	Intragastric ethanol infusion
iPSCs	Induced pluripotent stem cells
KCs	Kupffer cells
LDC	Lieber–DeCarli
LPS	Lipopolysaccharide
MACS	Magnetic activated cell sorting
MAP	Mean arterial pressure
NAFLD	Non-alcoholic fatty liver disease
NASH	Non-alcoholic steatohepatitis
NEMO	Nuclear factor (NF)-κB essential modulator
NIAAA	Chronic-plus-binge alcohol feeding model
PAPPA	Pregnancy-associated plasma protein A
PP	Portal pressure
PVBF	Portal vein blood flow
SMABF	Superior mesenteric artery blood flow
WD	Western diet
