# Supplementary material for: Recent Advances in Practical Methods for Liver Cell Biology: A Short Overview
Source: Int J Mol Sci. 2020 Mar 16;21(6):2027. doi: 10.3390/ijms21062027 (PMC7139397; doi:10.3390/ijms21062027)
Supplement: Supplementary file 1 [file ijms-21-02027-s001.pdf]

Supplementary Table S1. Most commonly used animal models for the study of liver diseases. Strength, Limitations and translatability to clinical studies.

Liver Disease	Animal Models	Strength	Limitations	Translatability to clinical studies	References
**ALD**	**A-DW model**	Easy to perform<br>Mild steatosis<br>Short- or long-term feeding	No liver fibrosis<br>Natural aversion in rodent	ALD animal models fail to replicate the all-round spectrum of human pathology<br>ALD rodent model would address: ethanol metabolism, oxidative stress presence, ROS production and immune system activation	[1–8]
	**LDC model**	Easy to perform<br>Steatosis<br>Short term feeding with no mortality rate	Mild mortality<br>No liver fibrosis<br>Low-level inflammation		
	**NIAAA model**	Cost and time efficient<br>High blood alcohol levels<br>Easy to perform<br>Steatosis<br>Liver injury<br>Inflammation	High mortality<br>No liver fibrosis<br>Represent only early alcoholic steatohepatitis		
	**LDC + "second hit" (LDC+CCl_4, LDC+DEN, LDC+ LPS, LDC+APAP)**	Easy to perform<br>Marked elevation of ALT and marked steatosis<br>Steatosis<br>Liver fibrosis	High mortality<br>Toxic components		
**NAFLD**	**Dietary models (HFD, HF-HC, MCD)**	Animals develop histologic and metabolic features of human NASH at 2-4 weeks	Lose weight<br>Diffuse fibrosis<br>No cirrhosis	Difference with human diet<br>Patients with NASH are not choline-deficient	[6,9–23]
	**Genetic models (ob/ob, fa/fa, SREBP-1c transgenic)**	Steatosis<br>Insulin resistance<br>Obesity	Modest fibrosis<br>~ 24 weeks to develop steatohepatitis without other insult<br>Severe and expensive	Metabolic and transcriptomic features in the liver are not totally in concordance to human NASH	
	**Combination therapies using chemicals (WD+CCl_4)**	Progressive steatosis<br>Insulin resistance<br>Fibrosis at 12 weeks<br>Cirrhosis<br>HCC at 24 weeks	Not represent human environment<br>Potential health hazards for its investigator		

Liver Disease	Animal Models	Strength	Limitations	Translatability	References
HCC	**Transplantation-based models (Xenograft, Isograft)**	Use of immune-deficient mice to avoid rejections	Cell-line derived xenografts lack the typical tumor cell heterogeneity<br>Unnatural environment for tumor growth	Immunological barrier produce rejections<br>Impossibility to study the immune system in tumor development	[4,6,19,24–28]
	**Chemically-induced models)<br>DEN**	Mimic the carcinogenesis produced from hepatotoxic compounds	Hepatotoxins produce particular liver lesions<br>Poorly reproducible<br>Slow tumor formation<br>Metastases are not observed	Reproduce the typical damage and episodes in the human cancer	
	**Combination therapies (DEN+CCL_4, WD+CCl_4)**	Obesity and simple steatosis in DEN+ CCL_4 model<br>Fibrosis by 12 weeks and HCC by 24 weeks in WD+CCl_4 model	Potential health hazards for its investigator	Hepatic transcriptomic alignment with human NASH	
Portal hypertension	**Partial portal vein ligation (PPVL)**	Easy surgery<br>Low mortality<br>Inexpensive<br>Reproducible<br>High PP after 2 days<br>Portal-systemic shunting and hyperdynamic circulatory changes	No fibrosis<br>No cirrhosis	Not all models express all disturbances characteristic of the portal hypertension syndrome	[17,29–33]
	**BDL**	Reproducible<br>Secondary biliary fibrosis and cirrhosis in 4–6 weeks<br>Periportal fibrosis after 1-3 weeks<br>50% ascites	10% Mortality rate in the first week<br>High mortality 7-10 weeks after induction<br>Not suitable for pharmacological studies with drugs that are eliminated through the biliary route<br>Mice develop marked dilation of the gallbladder after bile duct ligation, which may lead to perforation and choleperitoneum due to the presence of gallbladder		
	**CCl_4**	Histological changes in centrolobular area by 4 weeks<br>Progressive increase in PP in parallel with fibrosis<br>Cirrhosis at week 16	Potential health hazards for its investigator<br>Differences in development of PH due to different sensitivity to CCl_4<br>Peritoneal injection may cause damage in peritoneum, liver and intestines		
**Liver Disease**	**Animal Models**	**Strength**	**Limitations**	**Translatability**	**References**

**Angiogenesis**	**Genetic models (retroviral, lentiviral, sh/siRNA knock-down, CRISPR/Cas9-based method)**	Efficient and practical knock-<br>out of genes	Time consuming<br>Expensive<br>Requires careful genotyping	Angiogenesis is only a therapeutic target for liver fibrosis	[34–37]

References

1. Guo, F.; Zheng, K.; Benedé-Ubieto, R.; Cubero, F.J.; Nevzorova, Y.A. The Lieber-DeCarli Diet-A Flagship Model for Experimental Alcoholic Liver Disease. *Alcohol. Clin. Exp. Res.* **2018**, *42*, 1828–1840.
2. Liquid Diet Technique of ethanol administration: 1989 update. *Alcohol Alcohol.* **1989**.
3. Bertola, A.; Mathews, S.; Ki, S.H.; Wang, H.; Gao, B. Mouse model of chronic and binge ethanol feeding (the NIAAA model). *Nat. Protoc.* **2013**, *8*, 627–637.
4. Roychowdhury, S.; Chiang, D.J.; McMullen, M.R.; Nagy, L.E. Moderate, chronic ethanol feeding exacerbates carbon-tetrachloride-induced hepatic fibrosis via hepatocyte-specific hypoxia inducible factor 1α. *Pharmacol. Res. Perspect.* **2014**, *2*, e00061.
5. Tsukamoto, H.; Reidelberger, R.D.; French, S.W.; Largman, C. Long-term cannulation model for blood sampling and intragastric infusion in the rat. *Am. J. Physiol. - Regul. Integr. Comp. Physiol.* **1984**, *16*.
6. Brol, M.J.; Rösch, F.; Schierwagen, R.; Magdaleno, F.; Uschner, F.E.; Manekeller, S.; Queck, A.; Schwarzkopf, K.; Odenthal, M.; Drebber, U.; et al. Combination of CCl4 with alcoholic and metabolic injuries mimics human liver fibrosis. *Am. J. Physiol. Gastrointest. Liver Physiol.* **2019**, *317*, G182–G194.
7. Brandon-Warner, E.; Schrum, L.W.; Schmidt, C.M.; McKillop, I.H. Rodent models of alcoholic liver disease: Of mice and men. *Alcohol* 2012, *46*, 715–725.
8. Lamas-Paz, A.; Hao, F.; Nelson, L.J.; Vázquez, M.T.; Canals, S.; del Moral, M.G.; Martínez-Naves, E.; Nevzorova, Y.A.; Cubero, F.J. Alcoholic liver disease: Utility of animal models. *World J. Gastroenterol.* 2018, *24*, 5063–5075.
9. Tacke, F. Targeting hepatic macrophages to treat liver diseases. *J. Hepatol.* 2017, *66*, 1300–1312.
10. Schierwagen, R.; Maybüchen, L.; Zimmer, S.; Hittatiya, K.; Bäck, C.; Klein, S.; Uschner, F.E.; Reul, W.; Boor, P.; Nickenig, G.; et al. Seven weeks of Western diet in apolipoprotein-E-deficient mice induce metabolic syndrome and non-alcoholic steatohepatitis with liver fibrosis. *Sci. Rep.* **2015**, *5*, 12931.
11. Handa, P.; Morgan-Stevenson, V.; Maliken, B.D.; Nelson, J.E.; Washington, S.; Westerman, M.; Yeh, M.M.; Kowdley, K. V. Iron overload results in hepatic oxidative stress, immune cell activation, and hepatocellular ballooning injury, leading to nonalcoholic steatohepatitis in genetically obese mice. *Am. J. Physiol. - Gastrointest. Liver Physiol.* **2016**, *310*, G117–G127.
12. Marí, M.; Caballero, F.; Colell, A.; Morales, A.; Caballeria, J.; Fernandez, A.; Enrich, C.; Fernandez-Checa, J.C.; García-Ruiz, C. Mitochondrial free cholesterol loading sensitizes to TNF- and Fas-mediated steatohepatitis. *Cell Metab.* **2006**.
13. Takahashi, Y.; Soejima, Y.; Fukusato, T. Animal models of nonalcoholic fatty liver disease/ nonalcoholic steatohepatitis. *World J. Gastroenterol.* 2012, *18*, 2300–2308.
14. Zhong, F.; Zhou, X.; Xu, J.; Gao, L. Rodent Models of Nonalcoholic Fatty Liver Disease. *Digestion* 2019.
15. Friedman, S.L.; Neuschwander-Tetri, B.A.; Rinella, M.; Sanyal, A.J. Mechanisms of NAFLD development and therapeutic strategies. *Nat. Med.* 2018, *24*, 908–922.
16. Endo-Umeda, K.; Makishima, M. Liver X Receptors Regulate Cholesterol Metabolism and Immunity in Hepatic Nonparenchymal Cells. *Int. J. Mol. Sci.* **2019**, *20*, 5045.
17. Königshofer, P.; Brusilovskaya, K.; Schwabl, P.; Reiberger, T. Animal models of portal hypertension. *Biochim. Biophys. Acta - Mol. Basis Dis.* 2019, *1865*, 1019–1030.
18. Baeck, C.; Wehr, A.; Karlmark, K.R.; Heymann, F.; Vucur, M.; Gassler, N.; Huss, S.; Klussmann, S.; Eulberg, D.; Luedde, T.; et al. Pharmacological inhibition of the chemokine CCL2 (MCP-1) diminishes liver macrophage infiltration and steatohepatitis in chronic hepatic injury. *Gut* **2012**, *61*, 416–26.
19. Tsuchida, T.; Lee, Y.A.; Fujiwara, N.; Ybanez, M.; Allen, B.; Martins, S.; Fiel, M.I.; Goossens, N.; Chou, H.I.; Hoshida, Y.; et al. A simple diet- and chemical-induced murine NASH model with rapid

progression of steatohepatitis, fibrosis and liver cancer. *J. Hepatol.* **2018**, *69*, 385–395.

20. von Montfort, C.; Matias, N.; Fernandez, A.; Fucho, R.; Conde de la Rosa, L.; Martinez-Chantar, M.L.; Mato, J.M.; Machida, K.; Tsukamoto, H.; Murphy, M.P.; et al. Mitochondrial GSH determines the toxic or therapeutic potential of superoxide scavenging in steatohepatitis. *J. Hepatol.* **2012**, *57*, 852–859.
21. Van Herck, M.A.; Vonghia, L.; Francque, S.M. Animal models of nonalcoholic fatty liver disease—a starter's guide. *Nutrients* 2017, *9*.
22. Longato, L. Non-alcoholic fatty liver disease (NAFLD): A tale of fat and sugar? *Fibrogenes. Tissue Repair* **2013**, *6*, 14.
23. Lindström, P. The physiology of obese-hyperglycemic mice [ob/ob mice]. *ScientificWorldJournal.* 2007, *7*, 666–685.
24. Liedtke, C.; Luedde, T.; Sauerbruch, T.; Scholten, D.; Streetz, K.; Tacke, F.; Tolba, R.; Trautwein, C.; Trebicka, J.; Weiskirchen, R. Experimental liver fibrosis research: Update on animal models, legal issues and translational aspects. *Fibrogenes. Tissue Repair* 2013, *6*.
25. Luedde, T.; Beraza, N.; Kotsikoris, V.; van Loo, G.; Nenci, A.; De Vos, R.; Roskams, T.; Trautwein, C.; Pasparakis, M. Deletion of NEMO/IKKgamma in liver parenchymal cells causes steatohepatitis and hepatocellular carcinoma. *Cancer Cell* **2007**, *11*, 119–32.
26. Smit, J.J.M.; Schinkel, A.H.; Elferink, R.P.J.O.; Groen, A.K.; Wagenaar, E.; van Deemter, L.; Mol, C.A.A.M.; Ottenhoff, R.; van der Lugt, N.M.T.; van Roon, M.A.; et al. Homozygous disruption of the murine MDR2 P-glycoprotein gene leads to a complete absence of phospholipid from bile and to liver disease. *Cell* **1993**, *75*, 451–462.
27. Tolba, R.; Kraus, T.; Liedtke, C.; Schwarz, M.; Weiskirchen, R. Diethylnitrosamine (DEN)-induced carcinogenic liver injury in mice. *Lab. Anim.* **2015**, *49*, 59–69.
28. Uehara, T.; Pogribny, I.P.; Rusyn, I. The DEN and CCl4 -Induced Mouse Model of Fibrosis and Inflammation-Associated Hepatocellular Carcinoma. *Curr. Protoc. Pharmacol.* **2014**, *66*, 14.30.1–10.
29. Klein, S.; Hinüber, C.; Hittatiya, K.; Schierwagen, R.; Uschner, F.E.; Strassburg, C.P.; Fischer, H.-P.; Spengler, U.; Trebicka, J. Novel Rat Model of Repetitive Portal Venous Embolization Mimicking Human Non-Cirrhotic Idiopathic Portal Hypertension. *PLoS One* **2016**, *11*, e0162144.
30. Königshofer, P.; Brusilovskaya, K.; Schwabl, P.; Podesser, B.K.; Trauner, M.; Reiberger, T. Invasive Hemodynamic Characterization of the Portal-hypertensive Syndrome in Cirrhotic Rats. *J. Vis. Exp.* **2018**.
31. Klein, S.; Rick, J.; Lehmann, J.; Schierwagen, R.; Schierwagen, I.G.; Verbeke, L.; Hittatiya, K.; Uschner, F.E.; Manekeller, S.; Strassburg, C.P.; et al. Janus-kinase-2 relates directly to portal hypertension and to complications in rodent and human cirrhosis. *Gut* **2017**, *66*, 145–155.
32. Hellerbrand, C. Hepatic stellate cells--the pericytes in the liver. *Pflugers Arch.* **2013**, *465*, 775–8.
33. Klein, S.; Schierwagen, R.; Uschner, F.E.; Trebicka, J. Mouse and Rat Models of Induction of Hepatic Fibrosis and Assessment of Portal Hypertension. *Methods Mol. Biol.* **2017**, *1627*, 91–116.
34. Mejias, M.; Garcia-Pras, E.; Tiani, C.; Miquel, R.; Bosch, J.; Fernandez, M. Beneficial effects of sorafenib on splanchnic, intrahepatic, and portocollateral circulations in portal hypertensive and cirrhotic rats. *Hepatology* **2009**, *49*, 1245–56.
35. Calderone, V.; Gallego, J.; Fernandez-Miranda, G.; Garcia-Pras, E.; Maillo, C.; Berzigotti, A.; Mejias, M.; Bava, F.-A.; Angulo-Urarte, A.; Graupera, M.; et al. Sequential Functions of CPEB1 and CPEB4 Regulate Pathologic Expression of Vascular Endothelial Growth Factor and Angiogenesis in Chronic Liver Disease. *Gastroenterology* **2016**, *150*, 982–97.e30.
36.

Maillo, C.; Martín, J.; Sebastián, D.; Hernández-Alvarez, M.; García-Rocha, M.; Reina, O.; Zorzano, A.; Fernandez, M.; Méndez, R. Circadian- and UPR-dependent control of CPEB4 mediates a translational response to counteract hepatic steatosis under ER stress. *Nat. Cell Biol.* **2017**, *19*, 94–105.

37. Coch, L.; Mejias, M.; Berzigotti, A.; Garcia-Pras, E.; Gallego, J.; Bosch, J.; Mendez, R.; Fernandez, M. Disruption of negative feedback loop between vasohibin-1 and vascular endothelial growth factor decreases portal pressure, angiogenesis, and fibrosis in cirrhotic rats. *Hepatology* **2014**, *60*, 633–47.
